# P-1460. Ion Channels as Targets of an Anti-Tick Vaccine

**DOI:** 10.1093/ofid/ofaf695.1646

**Published:** 2026-01-11

**Authors:** Jacob F Myers, Matthias Schnell

**Affiliations:** Thomas Jefferson University, Philadelphia, PA; Thomas Jefferson University, Philadelphia, PA

## Abstract

**Background:**

Background- Ticks vector numerous diseases and are a growing public health concern as the incidence of tick vectored disease continues to increase. Tick vectored disease is heterogenous, limiting the impact of disease specific approaches and making the control of ticks themselves an attractive solution. Traditional chemical approaches to vector control are facing increasing challenges. When ticks ingest host blood, they also ingest antibodies, and if these antibodies bind to and disrupt these ion channels then vaccination could turn animals into walking reservoirs of a biologic acaricide as part of a One Health approach to preventing human disease.

**Methods:**

Methods- We aim to design a vaccine that induces immunity that kills ticks when they feed on a vaccinated host. We’ve identified the Glutamate Gated Chloride Channel (GluCl), the target of Ivermectin, as an ideal vaccine target. Previous studies showed that antibodies against GluCl could inhibit the GluCl and kill mosquitos when ingested with a bloodmeal. However, GluCl antibodies were limited by needing to “leak” out of the gut to reach their targets. Ticks are an ideal system to study GluCl vaccination because tick guts are orders of magnitude “leakier” to antibodies than mosquitos and ticks imbibe more blood.

**Results:**

Results- We designed and characterized a peptide-based vaccine against GluCl. We vaccinated mice with this candidate vaccine and recorded strong antibody reactions against GluCl. We microinjected ticks with 1) Ivermectin as a positive control or purified polyclonal IgG in PBS from 2) mice given a negative control vaccination, or 3) mice vaccinated with GluCl peptides. Tick mortality was 1) 84.8% 2) 31.3%, and 3) 90.6%, respectively. Fisher’s Exact test analysis of GluCl vs Negative Control yields a strongly significant effect (p=< 0.0001) while GluCl vs. Positive Control is non-significant (p=0.70).

**Conclusion:**

Conclusions- We have successfully shown that antibodies against an ion channel like the GluCl can exert a toxic effect in ticks. Future work will seek to explore the impact of these antibodies following natural feeding on a vaccinated animal and the potential of these approaches to interrupt cycles of disease transmission.

**Disclosures:**

All Authors: No reported disclosures

